# From Missed Diagnosis to Optimal Outcomes: A Comprehensive Review of Lisfranc Injuries

**DOI:** 10.7759/cureus.94615

**Published:** 2025-10-15

**Authors:** Ahmed Mohamed, Alaa Elasad, Abed Ullah Khan, Usman Fuad, Peter Salib

**Affiliations:** 1 Trauma and Orthopaedics, Royal Cornwall Hospital, Truro, GBR; 2 General Practice, Zagazig University, Zagazig, EGY; 3 Spinal Surgery, Neurosciences, Leeds Teaching Hospitals NHS Trust, Leeds, GBR; 4 Emergency Medicine, Norfolk and Norwich University Hospitals NHS Foundation Trust, Norwich, GBR

**Keywords:** foot anatomy, foot arthrodesis, lisfranc fracture dislocations, lisfranc sport injury, midfoot injury

## Abstract

Lisfranc injuries are traumatic disruptions of the tarsometatarsal joint complex. Injuries range from subtle ligamentous injuries to significant fracture dislocations. These relatively uncommon injuries are frequently missed during the initial presentation in emergency departments. Delayed or inadequate treatment can lead to chronic pain, midfoot arthritis, and significant functional disability. Early recognition and appropriate management of Lisfranc injuries are crucial for achieving optimal outcomes. Treatment strategies range from conservative management for stable injuries to surgical intervention for displaced or unstable injuries.

## Introduction and background

Lisfranc injuries represent 0.2% of all fractures, with an incidence of approximately one in 55,000 persons annually [1]. Despite their relative rarity, they are very significant, as missing them causes significant functional consequences. The Lisfranc joint complex was first described in the early 1800s by Jacques Lisfranc de St. Martin, a French surgeon in Napoleon's army [2]. It consists of the articulations between the five metatarsal bases, the three cuneiforms, the cuboid, and the complex ligamentous network that stabilizes them.

The demographic distribution of Lisfranc injuries shows a bimodal pattern. High-energy injuries predominantly affect males aged 20-40 years, often resulting from motor vehicle accidents or industrial trauma. Low-energy injuries demonstrate a more equal gender distribution and commonly occur in athletes, particularly in football players (29%), gymnasts, and dancers. The incidence in collegiate football players is approximately 4% annually, with offensive linemen at the highest risk due to the specific loading patterns during engagement [3-7].

Risk factors for Lisfranc injuries include participation in cutting sports, obesity (which increases midfoot stress during ambulation), and anatomical variations, such as a shallow mortise of the second metatarsal base. Diabetic neuropathy is a significant risk factor and a major cause of delayed presentation and is associated with poor outcomes, as decreased proprioception and protective sensation can lead to delayed diagnosis and treatment [8-10].

Understanding the mechanism of injury is important for suspecting injury when the patient describes it. Injuries can result from direct or indirect trauma. Direct trauma usually involves high-energy injuries, such as motor vehicle accidents and falls from heights. Indirect injuries are complex and require a cascade of rotational forces and axial loading with the foot in hyperplantar flexion. This creates a hyperflexion, compression, and abduction moment exerted on the forefoot that is transmitted to the tarsometatarsal (TMT) articulation, resulting in displacement of the metatarsals in the dorsal and lateral directions [11,12].

Literature has shown that approximately 20%-40% of Lisfranc injuries can be missed due to inadequate diagnostic investigation, lack of awareness by clinicians, and patients not seeking medical advice [13]. Diagnosing minor Lisfranc injuries requires a high index of clinical suspicion when patients present with foot trauma. In minor or pure ligamentous injuries, non-weight-bearing radiographs fail to show the injury, and weight-bearing radiographs are necessary; therefore, combining clinical sense with radiological findings is crucial for early management. Missed Lisfranc injuries result in irreversible and severe consequences that are difficult to manage. These consequences include chronic foot pain, declining ability to bear weight, forefoot abduction, altered gait patterns, instability during push-off, and flatfoot deformity [14]. This review aims to provide clinicians with a comprehensive understanding of Lisfranc injuries, including their clinical presentation, classification, diagnostic approaches, and evidence-based management strategies for acute and chronic presentations.

## Review

Anatomy

**Figure 1:**
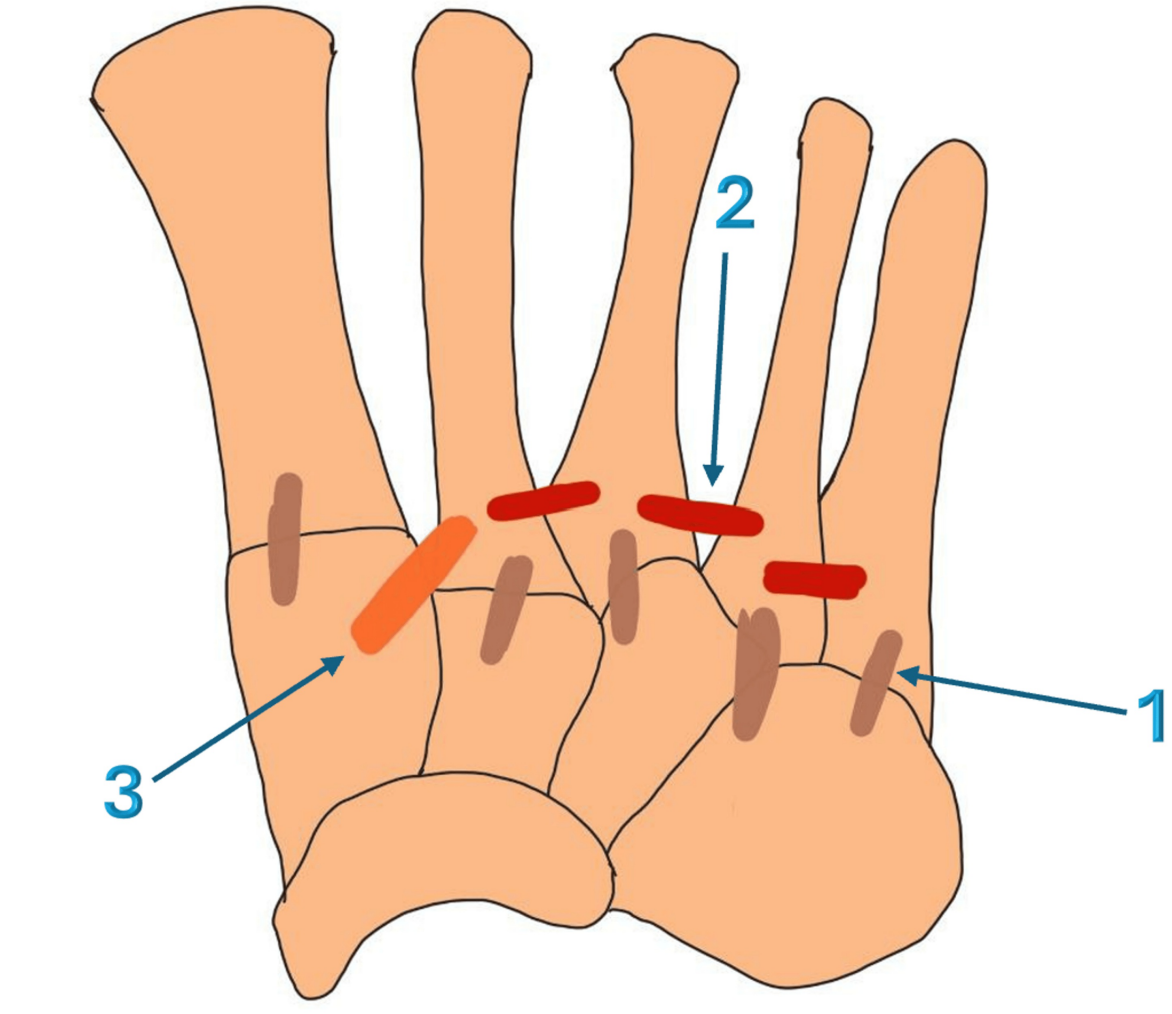
Ligamentous network of the midfoot This figure has been created using the Infinite Painter application and annotated using Microsoft Office 365 by author Alaa Elasad. 1 - Tarsometatarsal ligaments in brown; 2 - Intermetatarsal ligaments in red; 3 - Lisfranc ligament complex in orange

**Figure 2:**
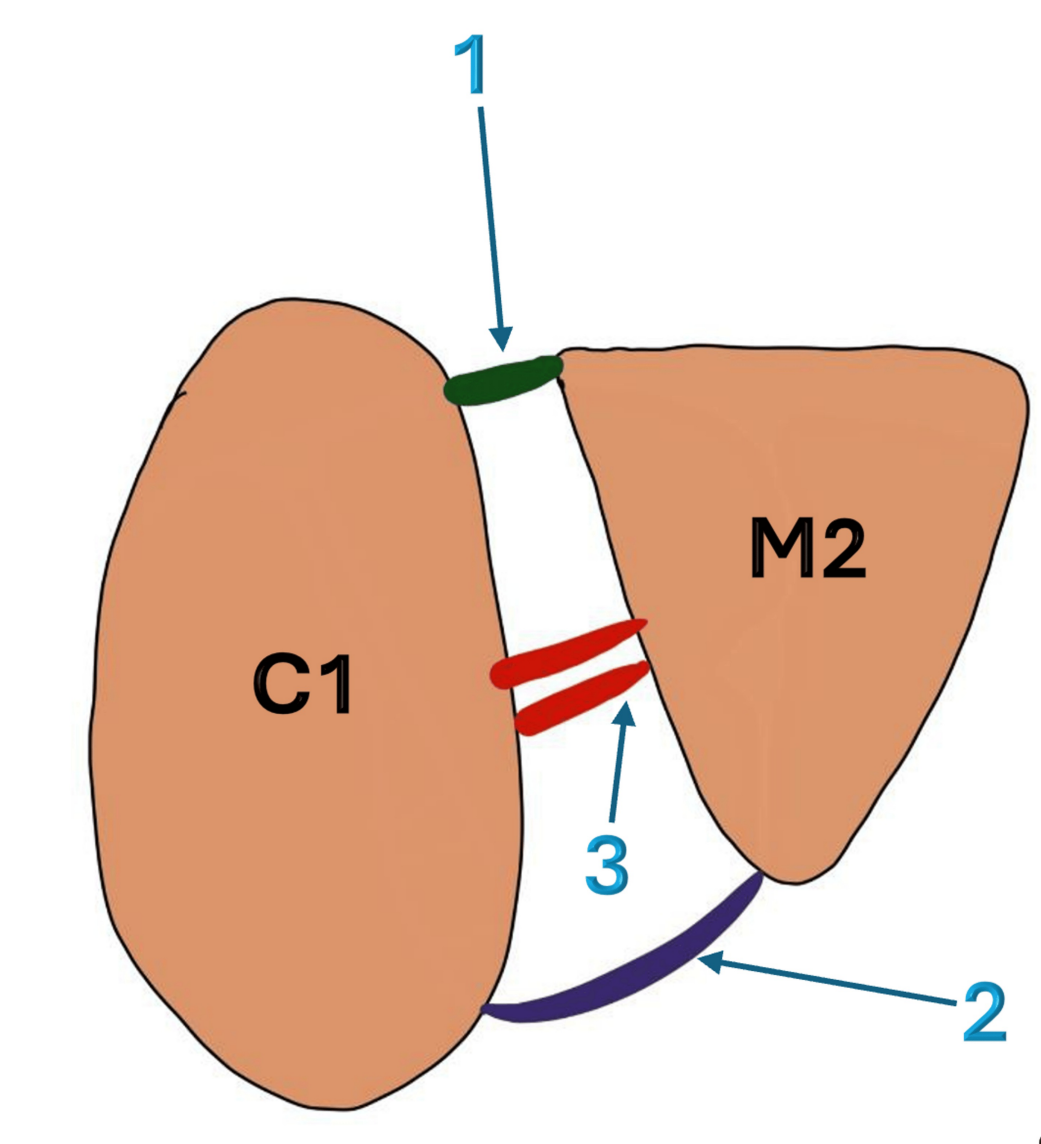
Anatomy of the Lisfranc complex This figure has been created using the Infinite Painter application and annotated using Microsoft Office 365 by author Alaa Elasad. C1 - Medial cuneiform; M2 - Second metatarsal; 1 - Dorsal tarsometatarsal (TMT) ligament; 2 - Plantar TMT ligament; 3 - Interosseous ligament (the Lisfranc ligament)

Ligamentous anatomy is crucial for understanding injury patterns. This ligamentous network consists of three main categories (Figure 1). (1) TMT dorsal and plantar ligaments crossing and stabilizing each TMT joint: The dorsal TMT joints are weaker, which explains the dorsal displacement of the metatarsals in Lisfranc injuries. (2) The intermetatarsal ligament that connects metatarsals from the second to the fifth: There is no intermetatarsal ligament between the first and second metatarsals; however, the Lisfranc ligament fills this gap, providing support and stability. (3) The Lisfranc complex is formed by the dorsal and plantar TMT ligaments, as well as the interosseous ligament, which is also called the Lisfranc ligament (Figure 2). The Lisfranc ligament extends from the medial cuneiform to the base of the second metatarsal. It is the strongest and thickest ligament [15].

Functionally, the midfoot is divided into three columns. The medial column includes the first TMT joint and provides primary weight-bearing support. The middle column consists of the second and third TMT joints. The lateral column consists of the fourth and fifth TMT joints and demonstrates the greatest mobility. The second metatarsal is the "keystone" of the transverse arch because it is set back within the mortise formed by the medial and middle cuneiform [16].

Biomechanics

The Lisfranc joint complex functions as a critical transition zone between the flexible hindfoot and the rigid forefoot during the gait cycle. During the stance phase, the midfoot must lock to create a rigid lever for an efficient push-off. This locking mechanism depends on the integrity of the Lisfranc ligament and the stability provided by the second metatarsal mortise. Force transmission through the midfoot follows predictable patterns, with approximately 30% of the body weight passing through the first ray and 20% through each of the lesser rays during normal gait [17,18].

The windlass mechanism helps keep the middle of the foot stable by lifting the arch when the heel rises. This occurs because of tension in the plantar fascia. If the Lisfranc complex is damaged, this mechanism stops working. This causes the arch to collapse and weakens the force transmission. Studies have shown that even a small shift of 2 mm in the TMT joints can reduce the strength of pushing off with the forefoot by up to 40%. This explains why untreated injuries can cause serious problems with movement [19,20].

Clinical presentation

Clinical presentation differs completely depending on the chronicity of the injury, as detailed in Table 1.

**Table 1:** Difference between acute and chronic presentation of Lisfranc injury This table is designed by author Ahmed Mohamed and not adapted from any previously published work. WB - Weight bear; TMT: Tarsometatarsal

Category	Acute presentation	Chronic presentation
Look	Unable to WB, midfoot swelling/wounds, plantar echymosis, obvious deformity (high energy)	Minimal or no swelling, forefoot abduction, flat foot deformity, altered gait pattern
Feel	Point tenderness, especially 1st and 2nd TMTJ, assess the compartments, and neurovascular assessment	Local TMT joints tenderness, dorsal osteophytes, plantar callus
Move	Piano test, pronation abduction test	Decreased midfoot flexibility, instability on push off

Acute presentation

In acute Lisfranc injuries, the clinical presentation can be divided into three main categories, namely, look, feel, and move, for completeness.

Look

A common early sign is the patient’s inability to bear weight. When inspecting the foot for suspected acute Lisfranc injuries, we should look for significant midfoot swelling or foot wounds that might indicate open fractures. Plantar ecchymosis at the midfoot is highly suggestive of Lisfranc injury. Obvious deformities may occur in cases of high-energy trauma.

Feel

Palpation of the foot reveals point tenderness over the TMT joints, particularly between the first and second metatarsal bases, which is the most consistent finding in Lisfranc injuries. Compartment syndrome should be carefully assessed and documented, especially in high-energy injuries. The examiner should assess for tense and swollen compartments and pain on passive stretch of the foot compartments (moving the toes) [21]. Neurovascular status must be documented, including dorsalis pedis and posterior tibial pulses, capillary refill time, and sensation over the superficial peroneal, deep peroneal, and tibial nerves. Motor function assessment can be limited by the pain level.

Move

Several provocative tests can help identify Lisfranc injuries. The piano key test, which involves dorsal-plantar stress on the patient’s metatarsals while stabilizing the midfoot, often elicits pain and may demonstrate instability. The pronation-abduction test, which involves passive pronation and abduction of the forefoot while stabilizing the hindfoot, reproduces pain in the TMT joint complex in ligamentous injuries.

Chronic presentation

Chronic Lisfranc injuries present weeks to years after the initial injury. Similarly, will discuss the chronic clinical picture using the “look, feel, and move” approach.

Look

Visual inspection in chronic cases may show minimal or no swelling as edema subsides over time. The most notable finding is the forefoot abduction deformity, which becomes apparent with weight-bearing due to longitudinal arch collapse, creating a flatfoot appearance. Avoidance of medial column loading due to pain forces patients to compensate through lateral foot overload. This compensation leads to subsequent altered gait patterns and lesser toe deformities.

Feel

Progressive arthritis of the TMT joints is common and may be evident as dorsal osteophytes palpable through the thin dorsal soft tissues. Plantar palpation may reveal calluses or areas of skin thickening corresponding to altered weight-bearing patterns.

Move

Range of motion testing in chronic injuries shows decreased midfoot flexibility. Patients often report instability or "giving way" of the foot during the push-off phase of gait.

Fracture classification

Myerson Classification

The Myerson classification, introduced in 1986, remains the most common system for classifying Lisfranc injuries [22]. This classification divides injuries into three main types based on the displacement pattern. Type A represents total incongruity and homolateral displacement of all five metatarsals in the same direction, either medially or laterally. Type B indicates partial incongruity and is subdivided into B1, representing medial displacement affecting the first metatarsal only (isolated medial column injury), and B2, indicating lateral displacement affecting one or more of the lateral four metatarsals (lateral column injury). Type C represents divergent patterns in which the first metatarsal is displaced medially, whereas the lateral metatarsals are displaced laterally.

Nunley and Vertullo Classification

In athletic injuries, subtle ligamentous injuries can have significant outcomes; thus, Nunley et al. proposed a classification system based on weight-bearing radiographs and clinical findings [7]. Stage I injuries show no displacement on weight-bearing radiographs, with a diagnosis based on clinical examination and possibly an MRI showing ligament sprain. Stage II injuries demonstrate 1-5 mm diastasis between the first and second metatarsals on weight-bearing radiographs, without loss of midfoot arch height. Stage III injuries show greater than 5 mm diastasis with loss of longitudinal arch height.

Diagnostic investigations

Plain Radiography

In high-energy injuries, where an obvious clinical deformity is present, non-weight-bearing radiographs are sufficient for injury detection and classification. In low-energy injuries or when significant clinical deformity is not present, standard radiographic evaluation should be weight-bearing anteroposterior (AP), lateral, and 30-degree oblique views of the affected foot. Non-weight-bearing films may miss up to 20%-40% of unstable, subtle injuries, making weight-bearing views essential when possible [13].

Key radiographic findings on the AP view include widening of the space between the first and second metatarsal bases. The "fleck sign," representing a small avulsion fragment in the first web space, indicates Lisfranc ligament dysfunction and is pathognomonic when present [23]. Loss of alignment between the medial border of the second metatarsal and the medial border of the middle cuneiform is another critical finding. The main radiographic finding in the oblique view is the alignment of the medial border of the fourth metatarsal with the medial border of the cuboid, as well as fractures of the metatarsal bases or cuneiforms that may not be apparent on other views. Lateral radiographs were evaluated for dorsal displacement of the metatarsal bases relative to the cuneiforms. When there is a clinical suspicion, weight-bearing radiographs of the contralateral foot can help compare the distance between the first and second metatarsal bases bilaterally [24].

Advanced Imaging

Computed tomography (CT) is indicated when plain radiographs are non-conclusive and when detailed assessment of the bony injury is needed for operative planning. Preoperative CT is important for surgical planning, particularly in comminuted injuries or complex fracture patterns. Three-dimensional (3D) reconstructions help identify all fracture fragments, assess articular involvement, and plan screw trajectories to avoid adjacent joint penetration. The surgeon should evaluate for associated injuries, such as cuboid fractures or intercuneiform instability, that may require additional fixation and can be easily missed on plain radiographs. Templates can be created preoperatively to determine the optimal implant size and position [25].

Magnetic resonance imaging (MRI) is the gold standard for evaluating pure ligamentous injuries and is particularly useful in athletes with suspected subtle injuries [26].

Dynamic ultrasound is an emerging investigation tool for evaluating Lisfranc ligament integrity, with studies showing sensitivity and specificity comparable to MRI for complete tears. Ultrasound can demonstrate widening of the C1-M2 interval with and without stress maneuvers. The advantages of ultrasound include its availability, cost-effectiveness, and ability to perform dynamic evaluation, although it requires a highly skilled radiologist to perform the correct technique and interpret the results [27].

Management

Acute Injury Management

Conservative treatment: It is reserved for stable injuries without displacement in weight-bearing views. Nunley et al.'s Stage I injuries in athletes may be managed non-operatively with non-weight-bearing in a short leg cast or boot for six weeks, followed by extensive physiotherapy before returning to full activities. Additionally, conservative treatment is usually offered to non-ambulatory or high-risk patients, such as those with poorly controlled diabetes with peripheral neuropathy and significant peripheral vascular disease, or patients with a high risk of wound complications, such as heavy smokers, alcohol abusers, or intravenous drug users [28,29].

Operative treatment: Surgical management is indicated for all unstable injuries with displacement greater than 2 mm, injuries with loss of arch height, and failed conservative treatment [30].

Optimal surgical timing is crucial for successful outcomes in Lisfranc injuries. In cases with significant soft tissue swelling, surgery may be delayed by 7-14 days to allow for swelling resolution. Waiting reduces the risk of wound complications and compartment syndrome. The "wrinkle test" indicates adequate swelling reduction for safe surgical intervention. It is performed by pinching the skin around the surgical approach, and the appearance of wrinkles indicates that the skin is elastic. During this waiting period, the foot should be elevated and immobilized in a well-padded posterior splint, maintaining the foot at 90° to prevent equinus contracture [31].

Intraoperatively, patient positioning and setup significantly impact the surgical ease and outcome. The patient should be positioned supine with a bump under the ipsilateral hip to internally rotate the leg and provide better access to the medial column. The foot should be extended beyond the table edge to facilitate fluoroscopic imaging in multiple planes. A radiolucent triangle or stack of towels under the ankle maintains the foot in a neutral position and facilitates lateral imaging. The preparation should extend from the toes to the knee, allowing for potential bone graft harvesting from the proximal tibia, if needed. Having both large and small fragment sets available is recommended, as screw sizes may vary based on the patient’s anatomy and bone quality [15,31].

Two primary surgical strategies are available for acute injuries.

(1) Open reduction and internal fixation (ORIF): It remains the gold standard for most acute Lisfranc injuries. The surgical principles include anatomic reduction of all affected joints, rigid fixation with screws or plates, and repair or reconstruction of the ligament, when possible [32].

The surgical approach for ORIF involves two dorsal incisions: one between the first and second metatarsals to address the medial and middle columns and another over the fourth metatarsal for the lateral column. The aims include achieving anatomical reduction and adequate fixation. Fixation options include transarticular screws (3.5 or 4.5 mm), dorsal bridge plating, and newer flexible fixation devices, such as suture buttons. The sequence of reduction should start from medial to lateral, fixing the TMT joint first, followed by securing the Lisfranc interval, and finally addressing the other metatarsals [15,33].

Recent biomechanical studies have shown that flexible fixation with suture button devices may allow physiologic micromotion while maintaining reduction. This method reduces the need for hardware removal. However, long-term clinical outcomes comparing rigid and flexible fixations remain under investigation [34,35].

(2) Joint arthrodesis: Primary arthrodesis is an evolving surgical procedure. Arthrodesis involves fusing joints by destroying the cartilage layer at the bone ends, exposing the bone, bone grafting, and fixing the joint components to heal as one unit. Proper alignment is crucial. The first metatarsal should be positioned in line with the medial cuneiform, maintaining an appropriate length and rotation. The medial and middle columns are fused, whereas the lateral column is preserved to maintain essential lateral column mobility. After achieving anatomic reduction, the joints are compressed and fixed with crossing lag screws or dorsal plating. This method has gained favor for certain injury patterns, particularly purely ligamentous injuries (as they take longer to heal compared to bony injuries), cases with significant comminution, and high-energy injuries with extensive cartilage damage. Multiple studies have demonstrated superior functional outcomes and quality of reduction with primary arthrodesis compared to ORIF for complete fracture dislocation (Myerson types A and C). Moreover, arthrodesis reduces the risk of a second operation in the previously mentioned scenarios, and metalwork removal is not necessary, compared to ORIF [36-38].

Postoperative protocols after acute injuries vary according to the fixation method and injury severity. Following ORIF, patients should remain non-weight bearing for six to eight weeks, followed by progressive physiotherapy-assisted weight-bearing in an ankle boot. Hardware removal, when necessary, is performed at four to six months postoperatively [28,39]. Return to sport is permitted at six to nine months for ORIF and 9-12 months for arthrodesis, based on functional testing and imaging confirmation of healing. Athletes should demonstrate single-leg hop testing at 90% of the contralateral side and pain-free, sport-specific movements before clearance [40-42].

Chronic Injury Management

The management of chronic Lisfranc injuries is challenging and often requires surgical intervention due to established deformities and arthritis. Conservative treatment for chronic injuries is generally palliative and includes custom orthotic devices with medial arch support and metatarsal pads, activity modification, anti-inflammatory medications, and corticosteroid injections for symptomatic arthritis. These measures may provide temporary relief but rarely result in long-term satisfaction [43].

Surgical treatment for chronic injuries involves midfoot arthrodesis, which remains the gold standard for chronic Lisfranc injuries with established arthritis or deformity. Studies have shown that patient satisfaction and functional outcomes following arthrodesis for chronic injuries range from 80% to 85% [44,45].

Complications

Complications following Lisfranc injuries are common and can be categorized as early or late. Early complications include wound dehiscence (5%-10%), superficial infection (3%-5%), and hardware failure (2%-4%). DVT occurs in approximately 1% of patients despite prophylaxis [46].

Late complications are more prevalent and include post-traumatic osteoarthritis (PTOA) (50%-70% at two years), chronic pain syndrome (25%-30%), and complex regional pain syndrome (5%-8%). Malunion with resultant flatfoot deformity occurs in 15%-20% of cases, particularly when the initial reduction is suboptimal. Hardware prominence requiring removal affects 30%-50% of ORIF patients, depending on implant choice. The development of adjacent joint arthritis, particularly affecting the midfoot and hindfoot, has been reported in long-term follow-up studies [1,26,47-49].

Outcomes and prognosis

Prognostic factors for optimal outcomes include anatomic reduction (the most critical factor), time to treatment (<3 weeks from injury), absence of associated injuries, and a low-energy injury mechanism. Poor prognostic indicators include non-anatomic reduction (>2 mm displacement), delayed treatment (>6 weeks), purely ligamentous injuries, and workers' compensation cases [50,51].

Functional outcome scores demonstrate variable results. The American Orthopaedic Foot and Ankle Society (AOFAS) midfoot scores average 75-85 points for anatomically reduced injuries versus 60-70 points for non-anatomic reductions. Return to pre-injury activity level is achieved in 70%-80% of patients with anatomic reduction, but only 30%-40% with non-anatomic reduction. Professional athletes have a 65% return-to-sport rate at the same level, with significantly better outcomes in those treated with primary arthrodesis for purely ligamentous injuries [41,52-54].

Patient education and counseling

Patient education is important for good results in Lisfranc injuries. Success depends on patients’ adherence to advice and understanding their condition. When first diagnosed, patients need to know how serious their injury is and how it should impact their lifestyle. Using models or images can help them understand why they need to avoid putting weight on their foot during their treatment time. Explaining that putting weight on the foot too soon can make the injury worse or can fail the operation often helps patients adhere to the treatment plan [55].

Setting realistic expectations is the cornerstone of patient counseling. Patients should understand that recovery takes 6-12 months, with maximal improvement taking up to two years. For surgical patients, discussing hardware prominence and the potential need for removal can prevent later dissatisfaction. Athletes require specific counseling about return-to-sport timelines and the possibility of not returning to their previous performance level, with studies showing only 65%-70% return to pre-injury levels in high-demand sports [56,57]. Work-related injuries warrant a discussion on potential permanent restrictions, particularly for jobs requiring prolonged standing or walking on uneven surfaces.

Long-term lifestyle modifications should be implemented early in the treatment process. Patients benefit from understanding that while most daily activities will be possible, high-impact activities may cause discomfort, even after successful treatment. Footwear modifications, including stiff-soled shoes or custom orthotics, may be permanent requirements. Weight management counseling is crucial, as an increased BMI correlates with poorer outcomes and increased midfoot stress. Patients should be educated about the warning signs of complications, including increasing pain, swelling, or deformity, which warrant immediate evaluation [58,59].

The psychological impact of prolonged recovery deserves attention in counseling. Many patients experience frustration with the extended non-weight-bearing period and slow functional recovery. Providing written materials, connecting patients with support groups, and scheduling regular follow-ups for questions can improve psychological outcomes. Documentation of all education provided is essential for medicolegal purposes, particularly given the high litigation rate associated with missed or inadequately treated Lisfranc injuries [60].

## Conclusions

Lisfranc injuries are complex and require careful evaluation and management to achieve satisfactory outcomes. Successful treatment starts with the correct diagnosis, even for minimal ligamentous injuries. This requires careful clinical examination and radiological investigation. Treatment options need to be chosen carefully, and a multidisciplinary team approach is required for optimal outcomes. Continued education regarding these injuries is essential to reduce the rate of missed diagnoses and improve the long-term outcomes for affected patients. Future research should be directed towards performing high-quality randomized controlled trials to compare different surgical options, especially in young athletes.
